# Evaluation of Extrusion Temperatures, Pelleting Parameters, and Vitamin Forms on Vitamin Stability in Feed

**DOI:** 10.3390/ani10050894

**Published:** 2020-05-20

**Authors:** Pan Yang, Huakai Wang, Min Zhu, Yongxi Ma

**Affiliations:** State Key Laboratory of Animal Nutrition, College of Animal Science and Technology, China Agricultural University, Beijing 100193, China; ypan23@163.com (P.Y.); huakaiwhk@cau.edu.cn (H.W.); s20173040486@cau.edu.cn (M.Z.)

**Keywords:** extrusion, pelleting, feed, vitamin stability, vitamin form

## Abstract

**Simple Summary:**

Since African Swine Fever is a pandemic in China, the Chinese feed mills implemented specific thermal processing to inactivate the virus. Farmers and animal nutritionists gradually focus on the destructive effects of feed processing on substances, e.g., vitamins, because vitamins are labile nutrients that are sensitive to the chemical and physical factors during thermal processing. The objectives of this study were to determine the effects of vitamin forms, extrusion temperature, and pelleting parameters on vitamin stability, and to determine which vitamins are destroyed by thermal processing. The deleterious impact of feed processing is of practical relevance to vitamin stability. The majority of B complex vitamins have great stability in feed processing, but the stability of fat-soluble vitamins was negatively affected by feed processing. In addition, microencapsulated vitamins had greater stability compared to non-microencapsulated vitamins. Based on the current results, decreasing the strength of feed processing and choice of suitable forms of the vitamin could be recommended in feed production.

**Abstract:**

Two experiments were conducted to determine the stability of microencapsulated and non-microencapsulated forms of vitamins in diets during extrusion and pelleting. We investigated the recovery of vitamins in swine diets after extrusion at 100 °C, 140 °C, or 180 °C. Next, two diets were conditioned at 65 °C (low temperature; LT) or 85 °C (high temperature; HT), and pellets were formed using a 2.5 × 15.0 mm (low length-to-diameter ratio; LR) or 2.5 × 20.0 mm (high length-to-diameter ratio; HR) die. The extrusion temperature had a significant effect on the recovery of vitamins E, B_1_, B_2_, B_3_, and B_5_ in the diets. The diet extruded at 100 °C had higher B_1_, B_2_, B_3_, and B_5_ vitamin recoveries than diets extruded at 140 °C and 180 °C. Microencapsulated vitamins A and K_3_ had greater stability than non-microencapsulated vitamins A and K_3_ at 100 °C and 140 °C extrusion. In the diet extruded at 180 °C, microencapsulated vitamins A, D_3_, and K_3_ had higher recoveries than non-microencapsulated vitamins A, D_3_, and K_3_. The recovery of vitamin K_3_ in diets after LTLR (low temperature + low length-to-diameter ratio) or HTLR (high temperature + low length-to-diameter ratio) pelleting was greater (*p* < 0.05) than after LTHR (low temperature + high length-to-diameter ratio) and HTHR (high temperature + high length-to-diameter ratio) pelleting. Our results clearly show that low extrusion temperature and low pellet temperature, and a low length-to-diameter ratio (L:D ratio) for pellet mill die are recommended for pig feed. Moreover, microencapsulated vitamins had greater stability compared to non-microencapsulated vitamins.

## 1. Introduction

Feed processing (sieving, grinding, extrusion, and pelleting) can lead to increased nutrient availability for an animal [1,2,3]. Specifically, pelleting has been shown to increase the average daily gain while decreasing the average daily feed intake for pigs [4]. It is widely accepted that pelleting improves average daily gain and feed efficiency in modern pig genotypes by 4%–8%, and the improvements are due to enhanced palatability, reduced waste, and the potential for improved nutrient utilization due to heat treatment of the ingredients [1,2,3]. Although thermal processes have variable effects on mycotoxins [5], the implementation of thermal processing can reduce microbial contaminations and biological hazards (i.e., the potential porcine epidemic diarrhea virus, *salmonella*) in feedstuffs [6,7]. Also, high-temperature extrusion is used frequently to improve feed hygiene and nutrient availability, as well as reduce anti-nutritional factors in feed [6,8]. Pelleting is the most prevalent processing in feed production. It has been demonstrated that pelleting increases feed quality, hygiene, and also reduces feed-carried pathogens [3,6,9]. Although extrusion and pelleting processes can improve feed hygiene, and the availability of some nutrients, the potential negative effects of these conditions should also be considered. Potential adverse effects include changes in lipid oxidation, protein denaturation, and cross-linking, starch gelatinization and dextrinization, degradation of vitamins and denaturation of enzymes, browning, and flavor formation [10,11]. Previous studies indicated that the stability of some vitamins could be as low as 50% when feeds are subjected to high-temperature processing [4,6,10].

Microencapsulated vitamins are currently available as a vitamin source, where the chemical structure of these vitamins is protected by microencapsulation [12]. It is believed that vitamin stability is much higher for microencapsulated vitamins post-feed processing because the protective wall materials of the microcapsules resist the heat, shear stress, and long periods of oxygen exposure during high-temperature processing. However, no information is available regarding the effect of microencapsulation on vitamin stability under different extrusion temperatures and pelleting parameters. Therefore, the objective of this study was to determine the effect of microencapsulation and extrusion or pelleting on the stability of commercially available vitamins in feed and to determine which vitamins are destroyed by thermal processing.

## 2. Materials and Methods

This study was conducted at the State Key Laboratory of Animal Nutrition at the China Agricultural University (Beijing, China). The extrusion of diets was conducted at the Institute of Food Science and Technology Chinese Academy of Agricultural Sciences (Beijing, China), and pelleting was completed at the China Agricultural University Feed Mill Educational Unit (Beijing, China). Approval from the Animal Care and Use Committee was not obtained for these experiments, because no animals were used.

### 2.1. Processing Parameters and Diets

Two experimental diets based on corn-soybean meal were used in this research. Diets were formulated to meet amino acid, vitamin, and mineral requirements for pigs according to the Nutrient Requirements of Swine [13]. Diet composition is shown in Table 1. The non-microencapsulated (NM) diet was formulated using non-microencapsulated vitamins, whereas the microencapsulated (M) diet was formulated with hydrogenated fat microcapsules containing microencapsulated vitamins, which have a coating structure and are made by the spray-drying technique. The processing parameters of the extrusion and pelleting for the NM and M diets are presented in Table 2.

### 2.2. Characterization of Non-microencapsulated and Microencapsulated Vitamins

Non-microencapsulated and microencapsulated vitamins were obtained from the Wellroad Animal Health Co. Ltd., China. High-resolution scanning electron microscopy (SEM) was used to observe the physical characteristics of non-microencapsulated and microencapsulated vitamins. The particle size distribution was measured with a series of 13 selected US standard sieves (Nos. 6, 8, 12, 16, 20, 30, 40, 50, 70, 100, 140, 200, and 270) and a pan, fitted into a sieve shaker. The sieving procedure was carried out according to a standard method [14]. In brief, 100 g of sample was sieved by shaking for 10 min. The mass of the material retained on each sieve, as well as that on the pan, was weighed and recorded. All measurements were performed in duplicate. The mass frequency (%) for material retained on each sieve size was calculated and plotted against each particle size category. The geometric diameter average (dgw) and geometric standard deviation (Sgw) were also calculated for each sieving replicate, based on the formula described in the ASAE Standards (2008) [14].

### 2.3. Extrusion Processing

In experiment 1 (Exp. 1), a 2 × 3 factorial design was used to evaluate the microencapsulated and non-microencapsulated vitamin forms and three extruder temperatures (100 °C, 140 °C, or 180 °C) on vitamin stability in the feed. The corn and soybean were ground to a mean particle size of 600 μm with a FAMSUN hammer mill (SFSP56 × 40C, FAMSUN, China). Vitamins from each product were initially mixed with 150 kg of corn. This premixing was completed to ensure proper mixing of the vitamins throughout the subsequent 400-kg batches of complete feed used for processing. Unprocessed samples were used to measure initial vitamin concentrations in the diet. The diets were extruded in a double screw annular gap extruder (DSE-25, Brabender Technologies GmbH & Co. KG, Germany) and extruded at 100 °C, 140 °C, or 180 °C. The screw diameter was 25 mm, and the pellet mill die length-to-diameter ratio (L:D ratio) was 20:1. These temperatures were selected because they are common parameters attainable in most commercial mills, and previous research exists for comparison.

The extrusion was repeated three times in three days, resulting in three replicates/temperature. At the beginning of the extrusion, flush feed containing no vitamins was used to warm the extruder to the first extrusion temperature. When the extruder barrel temperature was stable, the NM diet was added to the hopper and delivered into the extruder barrel by the feeder. After the NM diet was extruded at 100 °C and removed from the barrel, 10 kg of flush diet was added to the hopper to flush the system. While the extruder was maintained at 100 °C, the M diet was added to the hopper for extrusion. When both diets had been extruded at 100 °C, the flush feed was added to the hopper, and the temperature was increased to 140 °C. We ensured the temperature was stable before adding experimental diets. The NM and M diets were processed through the extruder (extruded at 140 °C or 180 °C) using the same procedures. The extruder was shut off between each run. These extrusion processes were repeated on days 2 and 3 to create replications.

### 2.4. Pelleting Processing

In experiment 2 (Exp. 2), a 2 × 4 factorial design was arranged with two diets (supplemented microencapsulated or non-microencapsulated vitamins) and four pellet parameters: two condition temperatures (65 °C vs. 85 °C) and two length-to-diameter ratios (L:D ratio, 6:1 vs. 8:1). The pelleting parameters were similarly selected because they are attainable variables in most commercial pellet mills, and previous research exists for comparison. The LTLR refers to low temperature + low L:D ratio, using a 15-mm-thick die with a 2.5 mm diameter hole at 65 °C. The LTHR refers to low temperature + low L:D ratio, using a 20-mm-thick die with a 2.5 mm diameter hole at 65 °C. The HTLR refers to a high temperature + low L:D ratio, using a 15-mm-thick die with a 2.5 mm diameter hole at 85 °C. The HTHR refers to a high temperature + low L:D ratio, using a 20-mm-thick die with a 2.5 mm diameter hole at 85 °C. Unprocessed feeds (mash) were prepared using the same procedure in Section 2.3. Steam conditioning (65 °C or 85 °C) of homogenized mixtures (NM diets or M diet) was carried out using a double-shaft steam conditioner (FAMSUN, SBTZ 10, China). Conditioning ended when the target temperature was achieved. After conditioning, the complete feed was pelleted in a pellet mill (FAMSUN MUZL 180, China) equipped with 15.0- or 20.0-mm-thick die having 2.5 mm openings.

The pelleting process was conducted on four consecutive days to create four replications. Flush feed containing no vitamins was used to warm the pellet mill equipped with 15.0-mm-thick die at the initial conditioning temperature. After the NM diet was pelleted (LTLR), 20 kg of flush diet was added to hopper to flush the system. While the pellet mill was still at 65 °C conditioning temperature, the M diet was added to the hopper. When the two diets had been processed at 65 °C using the 15.0-mm-thick die with 2.5 mm openings, the flush feed was added to the hopper, and the temperature was increased to 85 °C. We ensured the mill temperature was stable before proceeding with additional experimental diets. When the pelleting of LTLR and HTLR was complete, the pellet mill was shut off, and the die was changed to 20.0-mm-thick with 2.5 mm openings. Flush feed containing no vitamin was used to warm the pellet mill at 65 °C. After 65 °C pelleting (LTHR) for the NM diet was finished, 20 kg of flush diet was added to hopper to flush the system. While the pellet mill was still at 65 °C, the M diet was added to the hopper. When the two diets had been processed at 65 °C, the flush feed was added to the hopper, and the temperature was increased to 85 °C. The pellet mill was completely shut off when all runs were finished. These pelleting processes were repeated on days 2–4 to create replications.

The extruded and pelleted diets were dried in a double pass and cross-flow dryer. Samples of the extruded and pelleted diets were collected and stored for several hours at room temperature (22 °C) until a stable temperature was achieved. Samples were ground in Retsch ZM200 Mill (Retsch GMBH, Haan, Germany) and stored at -20 °C until analysis. The proximate and vitamin analyses were completed on all samples.

### 2.5. Chemical Analyses of Ingredients

Proximate analysis and analysis of vitamins in all feed ingredients were conducted at the Ministry of Agriculture and Rural Affairs Feed Potency and Safety Supervision and Testing Center located at the China Agricultural University, Beijing, China. Dry matter (DM) content of diets was determined by drying 5 g of sample in a forced-air oven (model GZX-9140 MBE; Boxun Company, Shanghai, China) at 105 °C to a constant weight (method 934.01) [15]. All diet samples were analyzed for crude protein (CP) (method 990.03) [15] using a Kjeldahl analyzer (Foss KjeltecTM 2100, Foss Kemao Inc., Beijing, China). Ether extract (EE) content in diet samples was determined using the petroleum ether extraction method (Method 920.39) [15] and an automated analyzer (Ankom XT15 Extractor; Ankom Technology, Macedon, NY, USA). Crude fiber (CF; Method 978.10) and ash (Method 942.05) were analyzed for all samples [15]. The nutrient composition of raw and thermally processed diets (Table 3) was confirmed. Diet nutrient concentrations were similar between raw and processed diets, except for the ether extract. In addition, samples were analyzed for vitamins A, D_3_, E, K_3_, B_1_, B_2_, B_3_, B_5_, and B_6_ content. Standards used were retinyl esters, cholecalciferol, α-tocopherol acetate, menadione, thiamine, riboflavin, pyridoxine, niacin, and pantothenic acid (Fluka, Sigma–Aldrich, Steinheim, Germany).

Vitamins A (VA) and E (VE) were determined by method 2012.10 [16]. In brief, feed samples were dissolved in 2% papain solution. Samples were placed in a 37 °C ± 2 °C water bath, and methanol was added to each sample tube for extraction. Vitamins A and E in this extract were analyzed by HPLC (Agilent 1200 Series; Agilent Technologies Inc., Santa Clara, CA, USA). For the extraction of vitamin D_3_ (VD_3_) from the feed, method 992.26 [16] was used. In brief, 5 g of sample was transferred to a centrifuge tube, and 15 mL anhydrous ethanol, 400 mg ascorbic acid, and 7.5 g KOH were added. The tube was placed in a 75 °C water bath. After saponification, the tube contents were extracted using ethyl ether/petroleum ether extraction. The supernatant was transferred to a clean tube and evaporated to dryness under nitrogen. Analysis of VD_3_ was completed using HPLC, followed by UV detection at 254 nm. For the determination of vitamin K_3_ (VK_3_), 5 g of feed was extracted with trichloromethane. The extract (1 mL) was transferred to an HPLC vial for direct injection. The injection volume was 10 μL, and UV absorbance was measured at 251 nm [17]. For the extraction of water-soluble vitamins (vitamins B_1_, B_2_, B_3_, B_5_, and B_6_) from diets, the procedure published by Chen et al. [18] was used. A total of 5 g of feed sample was extracted with 10 mM phosphate buffer and sonication in the dark. The supernatant was filtered through 0.1 µm polytetrafluoroethylene syringe filters. The extracted sample was analyzed using a 250 × 4.5 mm, 5 μm, Eclipse Plus C18 column (Agilent Technologies Inc., USA) on an Agilent liquid chromatography-tandem mass spectrometer (electrospray ionization source). Methods for analyzing vitamin concentration in samples were validated for repeatability, between-day precision, long-term precision, limits of quantitation, and linearity (data not shown) by the staff of the Ministry of Agriculture and Rural Affairs Feed Efficacy and Safety Evaluation Center in Beijing, China. The expected and analyzed vitamin contents found in diets NM and M are shown in Table 4. Although there is no published, accepted standard for vitamin recovery in animal feed, the analysis showed the experimental diets were within 10% of their formulated targets, which is consistent with the acceptable analytical variation. True recovery of vitamins through the feed processing methods was calculated using the following equation [19]: %True recovery = nutrient content per gram of processed feed × feed weight (gram) after processing/ (nutrient content per gram of raw feed × feed weight (gram) before processing) × 100.

### 2.6. Vitamin Stability Ranking

For each vitamin form, the stability was ranked based on the percent of each vitamin retained and a previously reported method [20]. The vitamin with the lowest stability at any of the three extrusion temperatures (100 °C, 140 °C, and 180 °C) or four pelleting parameters (low temperature + low L:D ratio, low temperature + high L:D ratio, high temperature + low L:D ratio, and high temperature + high L:D ratio) was ranked number one. Alternatively, the vitamin with the highest stability in any of those processing parameters was ranked number nine. Based on each vitamin stability rank order at different extrusion temperatures (100 °C, 140 °C, and 180 °C), the average vitamin stability rank order was calculated to represent the overall vitamin stability rank for extrusion. Also, based on each vitamin stability rank order at different pelleting parameters (low temperature + low L:D ratio, low temperature + high L:D ratio, high temperature + low L:D ratio, and high temperature + high L:D ratio), the average vitamin stability rank order was calculated to represent the overall vitamin stability rank for pelleting. Based on each vitamin’s overall stability rank order for extrusion and pelleting, the average vitamin stability rank order was calculated to represent the overall vitamin stability rank for feed processing.

### 2.7. Statistical Analysis

The normality of data was verified using the UNIVARIATE procedure of SAS (SAS Institute, Cary, NC, USA). The BOXPLOT procedure of SAS was used to check for outliers. Data were analyzed using the Proc Mixed procedure of SAS. The data from the extrusion and pelleting experiments were analyzed as completely random designs in 2 × 3 and 2 × 4 factorial arrangements, respectively, for the vitamin premix form and feed processing. The vitamin premix form, feed processing, and their interaction served as fixed variables. Only the main effects were discussed for responses in which the interaction was not significant, whereas contrasts were discussed where a significant interaction was detected. The LSMEANS statement was used to calculate treatment means. Significantly different means were identified using Tukey’s test. The results were considered significant at *p* < 0.05.

## 3. Results

**Figure 1:**
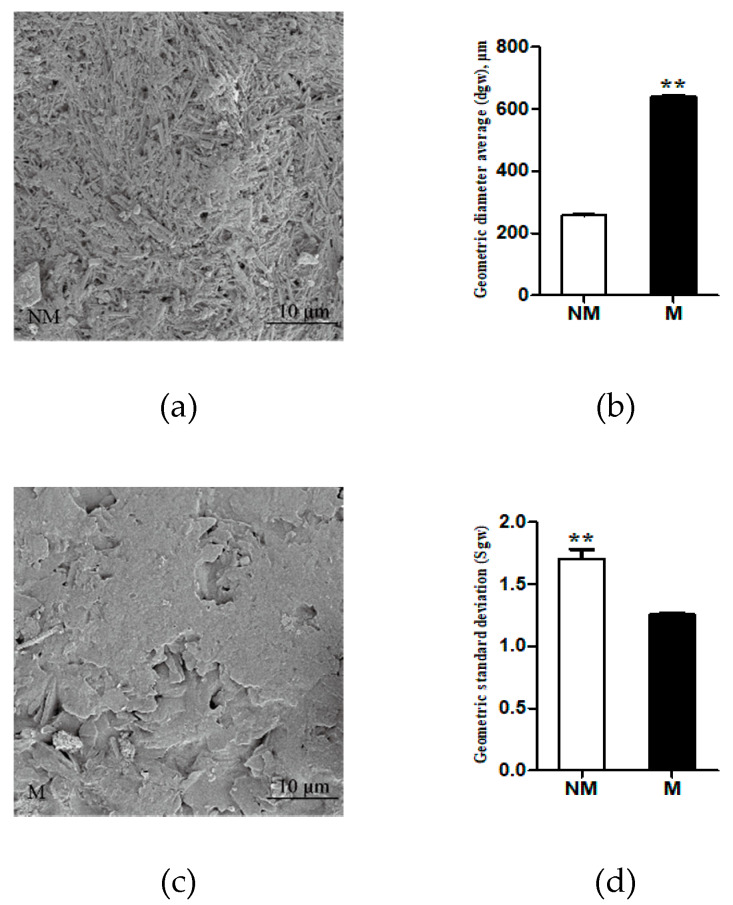
(**a**) The physical characteristics of non-microencapsulated vitamin form high-resolution scanning electron microscopy. (**b**) The geometric diameter average (dgw) of NM (non-microencapsulated) and M (microencapsulated) vitamins. (**c**) The physical characteristics of microencapsulated vitamin form high-resolution scanning electron microscopy. (**d**) The geometric standard deviation (Sgw) of NM (non-microencapsulated) and M (microencapsulated) vitamins. ** represent *p* < 0.01.

As shown in Figure 1, the physical analysis revealed that the average geometric diameter (dgw) of microencapsulated (M) vitamins was higher than non-microencapsulated (NM) vitamins. Regarding the geometric standard deviation (Sgw), microencapsulated vitamins were superior to their non-microencapsulated forms.

There was significant form × temperature interaction for vitamin A (VA), vitamin D_3_ (VD_3_), and vitamin K_3_ (VK_3_) recoveries (Table 5). At 100 °C and 140 °C extrusion temperatures, the diet containing the microencapsulated vitamins had higher recoveries of VA and VK_3_ than the diet containing the non-microencapsulated vitamins. At 180 °C extrusion temperature, the diet containing microencapsulated vitamins had higher VA, VD_3_, and VK_3_ recoveries than the diet containing non-microencapsulated vitamins (*p* < 0.05). The NM Diet, which contained the non-microencapsulated vitamins, had higher VA and VD_3_ recoveries at 100 °C extrusion than at 140 °C and 180 °C extrusion (*p* < 0.05), and the NM diet had higher VA and VD_3_ recoveries at 140 °C extrusion than at 180 °C extrusion (*p* < 0.05). However, the NM diet had lower VK_3_ recovery at 100 °C and 140 °C extrusion than at 180 °C extrusion (*p* < 0.05). The 100 °C extrusion conditions resulted in higher VA, VD_3_, and VK_3_ recoveries in diets containing the microencapsulated vitamins than at 140 °C and 180 °C extrusion (*p* < 0.05). In addition, the M diet had higher VA, VD_3_, and VK_3_ recoveries at 140 °C extrusion than at 180 °C extrusion (*p* < 0.05).

There was no significant form × temperature interaction for vitamin E, vitamin B_1_, vitamin B_2_, vitamin B_3_, and vitamin B_6_ (see Table 5). Vitamin forms had a significant effect on vitamin B_1_, vitamin B_2_, vitamin B_3_, and vitamin B_6_ recoveries. The degradation of vitamins B_1_, B_2_, B_3_, and B_6_ in the microencapsulated form was less (*p* < 0.05) than the non-microencapsulated form. Additionally, the extrusion temperature had a significant effect on vitamin E, vitamin B_1_, vitamin B_2_, vitamin B_3_, and vitamin B_5_ recoveries. No significant differences were detected among vitamin B_1_, vitamin B_2_, vitamin B_3_, and vitamin B_5_ recoveries in diets at 140 °C or 180 °C extrusion. At the 100 °C extrusion temperature, diets had higher recoveries of vitamin B_1_, vitamin B_2_, vitamin B_3_, and vitamin B_5_ than at 140 °C and 180 °C extrusions. Interestingly, there was no significant difference in the recovery of vitamin E at 100 °C and 180 °C extrusion, but diets at 140 °C extrusion had the lowest vitamin E recovery.

### 3.1. Effects of Pelleting on the Stability of Vitamins in Exp. 2

The recovery of vitamins after pelleting is shown in Table 6. There was no significant form × processing interaction on vitamin recovery. Moreover, vitamin form and processing had no significant effect on vitamin D_3_, vitamin E, vitamin B_2_, vitamin B_3_, vitamin B_5_, and vitamin B_6_ recoveries. Microencapsulation had a significant effect on the recoveries of vitamin A, vitamin K_3_, and vitamin B_1_. The recoveries of microencapsulated vitamin A, vitamin K_3_, and vitamin B_1_ were higher (*p* < 0.05) than their non-microencapsulated forms. Processing had a significant effect on vitamin K_3_ recovery. The recovery of vitamin K_3_ in LTLR (low temperature + low L:D ratio) and HTLR (high temperature + low L:D ratio) was greater than in LTHR (low temperature + high L:D ratio) and HTHR (high temperature + high L:D ratio) (*p* < 0.05).

### 3.2. Ranking of Vitamins

Vitamin stability was ranked to identify those vitamins that may be at greater risk of degradation during feed processing and could be used as indicators of vitamin quality in mixed diets. The vitamins were ranked to evaluate vitamin stability during pelleting and extrusion. Vitamin rankings are shown in Table 7 and Table 8. Non-microencapsulated vitamin K_3_ exhibited the greatest loss at all the three extrusion temperatures (100 °C, 140 °C, and 180 °C) and the four pelleting processes (LTLR, low temperature + low L:D ratio; LTHR, low temperature + high L:D ratio; HTLR, high temperature + low L:D ratio; HTHR, high temperature + high L:D ratio). Thus, vitamin K_3_ was ranked 1, whereas non-microencapsulated vitamin B_3_ was ranked 9 for all extrusion temperatures (Table 7). The non-microencapsulated vitamin B_5_ had the greatest stability in LTLR pelleting and was ranked 9. The non-microencapsulated vitamin B_6_ had the highest stability in LTHR pelleting and was ranked 9. Non-microencapsulated vitamin B_2_ had the greatest concentration in HTLR and HTHR pelleting and ranked 9 for these pelleting conditions. With respect to the microencapsulated forms, vitamin K_3_ exhibited the greatest loss at all three extrusion temperatures and four pelleting processes, and was consistently ranked 1. Conversely, vitamin B_2_ showed the greatest stability at 100 °C and 180 °C extrusion and HTLR and HTHR pelleting processes. It was ranked 9 (Table 8). Vitamin B_6_ at 140 °C extrusion had the highest stability and was ranked 9. Vitamin A in LTLR pelleting exhibited the greatest stability and was ranked 9. Vitamin B_5_ in LTHR pelleting showed the lowest degradation and was ranked 9. Based on the vitamin ranking tables we used, vitamin A, vitamin D_3_, vitamin K_3_, and vitamin B_1_ are the most easily degraded vitamins during feed processing, regardless of vitamin form.

## 4. Discussion

There were no differences in the analyzed nutrients except the ether extract between the mash and thermally processed treatments. A decrease in the recovery of ether extract after expanding has been reported in several studies [21,22], which was explained by the hydrothermal degradation of fat and by the formation of amylose-lipid complexes, which could not be extracted by ether. Vitamin supplementation of swine diets became essential when pig production moved into complete confinement housing. In confinement systems, pigs have no access to vitamin-rich pasture and are housed on slatted concrete floors, which limit the consumption of vitamins found in feces. Consequently, supplemental vitamins now play a vital role in meeting the vitamin requirements of pigs. Vitamin A, vitamin E, vitamin B_2_, vitamin B_3_, and vitamin B_5_ are commonly marginal or deficient in corn-soybean diets [11]. Compared to vitamin B_2_, vitamin B_3_, and vitamin B_5_, vitamins A and E appeared to be readily degraded under the varied processing conditions used in the study; thus, there is a high risk of insufficient vitamins A and E in manufactured diets.

The geometric diameter average (dgw) and geometric standard deviation (Sgw) are the average particle size of a sample and particle size variation, respectively. Microencapsulated vitamins have larger particle size and greater uniformity than their non-microencapsulated forms. Vitamin uniformity is critical because it will be further diluted hundred-fold in feeds; the difference in particle size between non-microencapsulated vitamins and feed constituents can give rise to the segregation of micro-ingredients. Also, high-resolution scanning electron microscopy revealed that the microcapsule wall material covered the vitamin crystal, protecting the vitamin in the feed matrix during processing.

### 4.1. Effects of Extrusion on Vitamin Stability 

Extrusion and pelleting have a positive impact on feed hygiene and nutrient availability. The extruded temperature under normal commercial mill conditions is above 90 °C. This high temperature is used frequently to produce more hygienic compounded feeds, specifically to reduce contamination with *Salmonella* and *Escherichia coli* [5,8]. The current data suggest that extrusion temperatures above 100 °C negatively affect vitamin recovery in mixed feed and are consistent with previous studies that heat processing of feed reduces vitamin stability [4,5]. The stability of vitamins depends on various conditions, such as temperature, oxidation, abrasion, and moisture. Our results revealed that feed processing (extrusion and pelleting) could reduce vitamin stability. A similar result has been previously observed from Charlton and Ewing [23] and Riaz et al. [24]. They concluded that higher extruded temperatures resulted in a low recovery of vitamins. Furthermore, it has been reported that the extrusion process has a negative impact on the degradation of vitamins A and E [25,26]. An investigation of high-heat cooking of foodstuffs resulted in a low recovery of vitamin D_3_ at 39%–45% [27], which corresponds well with our results. The chemical structures of vitamin A and vitamin D_3_ (the hydroxy group and double bonds) make them susceptible to oxidation during the feed processing. In addition, mash feed absorbs water vapor and heat during extrusion or pelleting, which decreases the stability of vitamin A and vitamin D_3_. This results in further destruction of vitamins A and D_3_ due to heightened oxidation reactions. This result is in agreement with a study by Tiwari and Cummins [28], who found that high-temperature, fast extrusion cooking decreased the stability of fat-soluble vitamins. Zielinski et al. [29] observed a significant decrease in vitamin E content of buckwheat during extrusion. Similarly, we observed that extrusion could cause a significant decrease in vitamin A, vitamin D_3_, vitamin E, and vitamin K_3_ contents of extruded diets.

We observed that water-soluble vitamins were stable during extrusion, but the recovery of vitamins B_1_ and B_6_ was lower than other B-complex vitamins. This result corresponds with several previous studies. Gadient and Fenster [30] reported that vitamin B_2_ in extruded feed was not affected by extrusion temperatures up to 150 °C. Furthermore, Li et al. [31] investigated the stability of B-complex vitamins in feeds extruded at 160 °C. They reported that vitamin B_2_, vitamin B_3_, and vitamin B_5_ had recovery rates of 100%, 96.3%, and 100%, respectively, but the recovery rates of vitamin B_1_ and vitamin B_6_ were 65.1% and 70.3%, respectively [31]. Marchetti et al. [32] reported the recovery rates of vitamin B_1_, vitamin B_2_, vitamin B_3_, vitamin B_5_, and vitamin B_6_ after extrusion at 96 °C as 87.89%, 86.05%, 92.17%, 85.84%, and 66.50%, respectively. The methylene bridge connecting the pyrimidine and thiazole moieties of vitamin B_1_ can easily be broken down during feed processing [10]. The thiazole moiety is less stable than the pyrimidine moiety and is easily cleaved by hydrolysis. Pyridoxine (vitamin B_6_) is commonly used for feed because pyridoxine is more stable than either pyridoxal or pyridoxamine [33], but pyridoxine is sensitive to light, particularly in neutral and alkaline solutions [11]. Major changes were observed in the levels of important water-soluble vitamins, including vitamins B_1_ and B_6_, which are regarded as the most heat-sensitive [34]. For B group vitamins, the thermostabilities of vitamins B_1_ and B_6_ were poor, and vitamin B_1_ showed the greatest reductions during processing. Vitamins B_2_, B_3_, and B_5_ are stable upon heating and not affected by processes such as hot air convection, infrared, high-pressure steam, or microwave during cooking [33]. Thermochemistry of B vitamins shows the molecules of vitamins B_2_, B_3_, or B_5_ are tightly bound and easily form hydrogen bonds, which improves their thermostability.

In addition, there are some extrusion parameters that increase vitamin destruction, e.g., screw rpm, moisture, energy input, etc. [34]. Generally, the recovery of the vitamin in extrusion decreases with increasing screw speed, increasing pressure in the barrel, decreasing moisture, and increasing energy input [24,26,34]. In the present study, pressure in the barrel at low-temperature treatment was greater than at high-temperature treatment, which may contribute to huge vitamins A, E, and K_3_ losses of more than 50% during extrusion. However, no serious vitamin degradation occurred in other vitamins. The reason may be explained that those vitamins are more sensitive to temperature than pressure. During extrusion, very complex reactions were ongoing; therefore, it is not easy to study the influence of each factor. On the other hand, vitamins differ greatly in chemical structure, and available form, their stability during extrusion also varies [24,34]. The degradation of most vitamins could be avoided by reducing the temperature and pressure in the extruder. 

### 4.2. Effects of Pelleting on the Stability of Vitamins

Pelleting of pig feed has been practiced for decades; the specific energy input in the pellet mill has increased due to harder pellets requested in the global market and used for cereal by-production [2,3,6]. Prolonged conditioning and double pelleting increase the aggressiveness of the pelleting process [35], which could compromise bioactive substances [36] and the stability of vitamins in diets [32,37]. After extrusion, the recovery of vitamins was generally lower than after pelleting. This observation is in line with Marchetti et al. [32], where the extrusion process involves much higher energy input, pressure, and temperatures than pelleting. Conditioning temperatures in pelleting from 65 °C to 85 °C and an L:D ratio of 6:1 to 8:1 are typical commercial mill conditions, depending on the type of diet. The current data suggest that conditioning temperatures above 65 °C and an increased L:D ratio can negatively affect the vitamin recovery of feed products. The resistance of vitamins A, D_3_, and E recoveries to increased temperature during pelleting is consistent with other reports. Jones [38] reported that the vitamin A concentration in feed was reduced by 6.5% by pelleting at 80 °C, and Cutlip et al. [39] observed that vitamin A lost 6.7% of its biological activity when pelleted at 93.3 °C. Furthermore, the concentrations of vitamins A and E decreased to 6.62% and 4.83% of their initial values in the samples of pelleted feed (60 °C, L:D ratio 6:1) [25,26], and pelleting temperatures of up to 88 °C did not affect the stability of vitamin D_3_ [40]. Modern vitamin production gives vitamin A and vitamin D_3_ greater protection against moisture, heat, and pressure during pelleting [11,26]. Commercial forms of vitamins A and D_3_ exist in the matrix as a cross-linked beadlet generally composed of gelatin [10]. The cross-linked beadlet also reduces the effect of shear force or pressure during pelleting. The antioxidant capabilities of the tocopherols, due to the free phenolic hydroxy group, compromise the stability of vitamin E acetate. Esterification with acetic acid eliminates its antioxidative nature, thereby improving stability in pelleting [10,11,33]. We observed that the recoveries of vitamin A, vitamin D_3_, and vitamin E in extruded diets were lower than pelleted diets, which was likely due to higher abrasion, heat, and pressure in extrusion than pelleting. Interestingly, the loss of vitamin K_3_ was over 50% in the present study, which is higher than the results of Marchetti et al. [32]. This result may be caused by prolonged conditioning times in our experiment, which increases the leaching of vitamin K_3_ and prolongs oxidation-reduction reactions. Additionally, the VK_3_ source used in the present study was menadione sodium bisulfite (MSB), which is most commonly used in the feed industry [11,12]. However, MSB has a high solubility in water, which led to increased leaching during feed processing. MSB also has limited stability to light, heat, humidity, and pressure [11,12,20], in which the molecule is destroyed.

Although there was no significant difference, the recovery of B complex vitamins at 85 °C was slightly higher than that at 65 °C. In addition, the recovery of water-soluble vitamins was slightly higher at low pressure (L:D ratio 6:1) than at high pressure (L:D ratio 8:1). The reason was due to the degradation of B vitamins that can easily occur at 65 °C, and this is consistent with Lewis et al. [40]. Kimura et al. [41] compared various methods of cooking pork, and found that vitamin B_1_ loss was the highest during boiling, followed by steaming, parching, and frying. This was explained by the leaching of vitamin B_1_ into water, due to its water-soluble nature. Vitamins B_3_ and B_6_ destruction occur during the precooking process, when they may leach into water but processing alone is not expected to destroy the vitamin [42]. However, cooking beef products to 57 °C internal temperature has demonstrated a loss of both vitamins B_2_ and B_3_ [43]. Vitamin B_5_ is also sensitive to cooking in water. Stability factors for vitamin B_5_ in legumes (33%–76%) were significantly influenced by the pre-soaking method and cooking times [42]. Those experiments were conducted using food materials and may not correspond to water-soluble vitamin degradation in feed nutrition, but the degradation of B vitamins can occur at low temperatures. To the best of our knowledge, there is limited information about vitamin stability data during the pelleting of swine feed. Due to the lack of water-soluble vitamin stability data during the pelleting of swine feed, data presented herein, and from other reports, on the stability of vitamins could be useful in modifying vitamin premixes.

### 4.3. Effects of Microencapsulation on Vitamin Stability During Extrusion and Pelleting

Microencapsulation is a process by which substances are coated with a continuous film of polymeric material [33]. The substance, if sensitive to oxygen, moisture, or light, can be stabilized by microencapsulation [12]. In the current study, the stability of microencapsulated vitamin A was higher than that of non-microencapsulated vitamin A regardless of the extruded temperature. The reason is likely because vitamin A contains four double bonds and one hydroxyl group, which are unstable under high temperature [11,26,34,37]. Microencapsulation can give crystalline vitamin A greater protection against oxidation reactions because the polymeric film provides effective protection of core material during processing [11,12]. Cutlip et al. [39] reported that diets containing vitamin A (in the form of retinyl acetate in a gelatin-lactose coating with antioxidants) exhibited only slight oxidative damage when pelleted at 93.3 °C. Extrusion and pelleting may cause a detrimental loss of vitamin D, but it depends on the heating process [10,11,24,34]. Our results show that the recovery rate of microencapsulated vitamin D_3_ was higher during extrusion at 180 °C, compared with non-microencapsulated vitamin D_3_. Microencapsulation can reduce the reactivity and incompatibility of compounds with the outside environment, enhancing their stability in conditions of heat, moisture, oxygen, among others [12,33]. Thus, the stability of vitamin D_3_ during feed processing can be greatly improved. In addition, the stability of the microencapsulated vitamin E was slightly higher than the non-microencapsulated vitamin E. During microencapsulation of vitamin E; it is esterified with acid, which provides protection and improves stability [10,11]. Microencapsulation significantly reduced the loss of vitamin K_3_ during extrusion and pelleting. The result was consistent with Marchetti et al. [32] who reported that the recovery of menadione from coated forms after extrusion or pelleting was significantly higher than non-coated forms.

There were no significant differences in the recoveries of vitamin B_2_, vitamin B_3_, and vitamin B_5_ when comparing the two forms of vitamins. The result is consistent with Riaz et al. [24], who reported that vitamin B_2_, vitamin B_3_, and vitamin B_5_ were stable during thermal processing. The recoveries of microencapsulated vitamins B_1_ and B_6_ were higher than their non-microencapsulated forms when extruded at 100 °C, 140 °C, and 180 °C. This is likely because vitamin B_1_ is highly unstable upon heating and will actively participate in the Maillard reaction during heat treatments [44]. Additionally, vitamin B_6_ rapidly degrades with increasing temperature. Microencapsulation likely protected vitamin B_1_ against the Maillard reactions caused by heat and pressure during extrusion and pelleting [44]. Microencapsulation could also minimize contact with carbonyl groups of reducing sugars. Increased thermal stability of microencapsulation was also observed by Chatterjee et al. [45]. 

To our knowledge, the present study is the first to establish vitamin ranking for the stability of various vitamins following extrusion and pelleting. This ranking could be used to identify vitamins that are vulnerable, and producers can analysis those vitamins for the feed manufacturing quality control program. Based on our vitamin ranking, vitamin A, vitamin D_3_, vitamin K_3_, and vitamin B_1_ are the top four sensitive vitamins under feed processing, regardless of vitamin form, and they could be used as indicators to determine overall dietary vitamin quality after feed manufacturing. We suggest monitoring the content of these vitamins in the manufacturing of feed.

## 5. Conclusions

The deleterious impact of feed processing, particularly extrusion, is of practical relevance to vitamin stability and feed quality. Our results clearly show a low extrusion temperature is recommended for pig feed. Based on the current results, we generally suggest that a reduction of pelleting strength, low temperature, and low L:D ratio may recommend for compound vitamin mixes used in the production of pig feed. Our research found that the majority of B complex vitamins have great stability in feed processing, but recovery of fat-soluble vitamins (vitamin A, vitamin D_3_, vitamin E, and vitamin K_3_), vitamin B_1_, and vitamin B_6_ was negatively affected by feed processing. Our results clearly indicate improved stability of microencapsulated vitamins, particularly vitamin A, vitamin D_3_, vitamin K_3_, vitamin B_1_, and vitamin B_6_.

## Figures and Tables

**Table 1:** Ingredient composition and calculated nutrients of the experimental diet (%, as-fed basis).

Ingredient	Diets ^1^	
	NM	M
Corn	68.81	68.81
Soybean meal	24.00	24.00
Monocalcium phosphate	1.60	1.60
Limestone	0.80	0.80
Salt	0.30	0.30
Soybean oil	2.75	2.75
L-lysine HCl	0.53	0.53
DL-methionine	0.13	0.13
L-threonine	0.24	0.24
Tryptophan	0.04	0.04
NMVP ^2^	0.30	-
MVP ^3^	-	0.30
Trace mineral premix ^4^	0.50	0.50
Total	100.00	100.00
Calculated values		
SID Lysine	1.23	1.23
SID Methionine	0.36	0.36
SID Threonine	0.74	0.74
SID Tryptophan	0.20	0.20
ME, kcal/kg	3383.56	3383.56
CP	17.1	17.1
Ca	0.72	0.72
P	0.60	0.60
Available P	0.41	0.41

^1^ NM, non-microencapsulated; M, microencapsulated. ^2^ NMVP = Non-microencapsulated vitamin premixes. NMVP provided the following per kilogram of feed: Vitamin A, 13,500 IU; vitamin D_3_, 3,000 IU; vitamin E, 30 mg; vitamin K_3_, 3 mg; vitamin B_12_, 24 µg; riboflavin, 6 mg; pantothenic acid, 18 mg; niacin, 30 mg; choline chloride, 400 mg; folacin, 0.12 mg; thiamine, 1.5 mg; pyridoxine, 3 mg; biotin 0.03 mg. ^3^ MVP = Microencapsulated vitamin premixes. MVP provided the following per kilogram of feed: Vitamin A, 13,500 IU; vitamin D_3_, 3,000 IU; vitamin E, 30 mg; vitamin K_3_, 3 mg; vitamin B_12_, 24 µg; riboflavin, 6 mg; pantothenic acid, 18 mg; niacin, 30 mg; choline chloride, 400 mg; folacin, 0.12 mg; thiamine, 1.5 mg; pyridoxine, 3 mg; biotin 0.03 mg. ^4^ Trace-mineral premixes provided per kilogram of diet: 80 mg Fe as iron sulfate, 60 mg Cu as copper sulfate, 17.5 mg Mn as manganese oxide, 65 mg Zn as zinc oxide, 0.3 mg I as ethylenediamine dihydroiodide, and 0.2 mg Se as sodium selenite.

**Table 2:** Processing parameters of experimental diets.

Parameters	Extrusion			Pelleting			
	100 °C	140 °C	180 °C	LTLR	LTHR	HTLR	HTHR
Machine type	DSE-25 ^1^	DSE-25	DSE-25	MUZL 180 ^2^	MUZL 180	MUZL 180	MUZL 180
Feeding rate, rpm	40	40	40	30	30	30	30
Screw speed, rpm	160	160	160	-	-	-	-
Condition time, s	-	-	-	60	60	60	60
Temperature intake, °C	100	140	180	65	65	85	85
Barrel pressure, MPa	5	2	1	ND	ND	ND	ND
Stream pressure, MPa	-	-	-	0.3	0.3	0.3	0.3
Diameter, mm	25	25	25	2.5	2.5	2.5	2.5
L:D ratio	20:1	20:1	20:1	6:1	8:1	6:1	8:1
Moisture content, %	25	25	25	12	12	12	12

ND = Not detected; L:D ratio, pellet mill dies length-to-diameter ratio; LTLR, low temperature + low L:D ratio; LTHR, low temperature + high L:D ratio; HTLR, high temperature + low L:D ratio; HTHR, high temperature + high L:D ratio. ^1^ DSE-25, extruder, Brabender Technologie GmbH & Co. KG, Germany. ^2^ MUZL 180, pellet mill, FAMSUN, China.

**Table 3:** Analyzed nutrient concentration in manufacturing productions (%, as-fed basis) ^1^.

Items ^2^	NM Diet ^2^									M Diet ^3^								
	Extrusion				Pelleting					Extrusion				Pelleting				
	Mash	Temperature, °C			Mash	65 °C		85 °C		Mash	Temperature, °C			Mash	65 °C		85 °C	
		100	140	180		6:01	8:01	6:01	8:01		100	140	180		6:01	8:01	6:01	8:01
DM	88.24	88.70	88.74	88.05	88.51	88.45	88.72	88.07	88.23	88.15	88.55	88.23	88.67	88.47	88.81	88.49	88.03	88.20
CP	17.13	17.19	17.38	17.03	16.96	17.11	17.36	17.09	17.28	17.16	16.93	17.38	17.52	16.93	17.39	16.97	17.26	17.20
CF	4.25	4.38	3.99	4.11	4.03	4.06	4.21	3.95	4.02	4.27	3.93	4.29	4.20	4.17	4.09	3.98	4.14	4.08
EE	6.41	2.63	2.77	2.90	6.37	6.41	6.58	6.49	6.41	6.32	2.79	2.98	3.17	6.41	6.22	6.79	6.82	6.45
Ash	4.72	4.31	4.40	4.59	4.99	4.61	4.73	4.51	4.61	4.83	4.80	4.53	4.27	4.81	4.85	4.57	4.6	4.49

^1^ Analyses were completed in duplicate according to the Association of Official Agricultural Chemists (AOAC) International official methods 934.01 (moisture), 984.13 (crude protein), 978.10 (crude fiber), 942.05 (Ash) and 920.39 (EE). ^2^ The NM diet was formulated using non-microencapsulated vitamins. ^3^ The M Diet was formulated with microencapsulated vitamins.

**Table 4:** Vitamin concentration in unmanufactured mash diet (as-fed basis) ^1^.

Item ^2^	NM Diet			M Diet		
	Calculated ^3^	Analyzed	Ratio ^4^	Calculated	Analyzed	Ratio
Vitamin A, IU/kg	13,500	13,660.08	101.19	13,500	13,784.01	102.10
Vitamin D_3_, IU/kg	3000	3084.27	102.81	3000	3115.35	103.85
Vitamin E, mg/kg	30	32.12	107.09	30	31.10	103.68
Vitamin K_3_, mg/kg	3	3.12	104.10	3	3.19	106.20
Vitamin B_1_, mg/kg	3	3.05	101.57	3	3.02	100.80
Vitamin B_2_, mg/kg	6	6.12	102.05	6	6.11	101.80
Vitamin B_3_, mg/kg	30	30.41	101.38	30	30.86	102.87
Vitamin B_5_, mg/kg	18	18.42	102.31	18	18.30	101.68
Vitamin B_6_, mg/kg	3	3.10	103.43	3	3.15	105.00

^1^ The NM diet was formulated using non-microencapsulated vitamins, whereas M diet was formulated with microencapsulated vitamins. ^2^ Values represent means of replicate samples each analyzed in duplicate (method 2012.10 for vitamin A (VA) and vitamin E (VE) analysis; AOAC 2012, method 992.26 for vitamin D_3_ (VD_3_) analysis; AOAC 2012, GB/T 18872-2017 for vitamin K_3_ (VK_3_) analysis; National standard 2017, the method for water-soluble vitamins analysis; Chen et al. [18], Ministry of Agriculture and Rural Affairs Feed Efficacy and Safety Evaluation Center, Beijing, CN). ^3^ Calculated values were determined from manufacturers guaranteed minimum. ^4^ Analyzed to calculated ratio.

**Table 5:** Effects of extruded temperature (Temp.) and vitamin forms (non-microencapsulated or microencapsulated) on the percentage of vitamins in diets (Experiment 1) ^1^.

Form	Temp.	VA	VD_3_	VE	VK_3_	VB_1_	VB_2_	VB_3_	VB_5_	VB_6_
NM	100 °C	46.34	73.94	47.34	7.17	94.91	98.71	102.59	99.19	74.35
	140 °C	34.75	53.14	42.23	6.12	79.24	87.77	89.75	84.32	78.12
	180 °C	30.76	40.26	45.17	11.64	78.31	90.16	87.18	85.51	71.82
M	100 °C	56.41	75.73	49.17	48.04	100.71	105.16	104.81	100.83	101.88
	140 °C	40.25	56.86	43.09	35.20	83.81	91.39	90.59	87.47	92.46
	180 °C	41.80	60.74	46.41	38.55	83.60	90.76	90.20	84.41	89.25
SEM		0.74	3.40	1.10	1.81	0.68	1.28	1.14	1.75	3.16
Main effects										
Form	NM	37.28	55.78	44.91	8.31	84.15 ^y^	92.21 ^y^	93.18 ^y^	89.67	74.76 ^y^
	M	46.15	64.44	46.22	40.60	89.37 ^x^	95.77 ^x^	95.20 ^x^	90.90	94.53 ^x^
Temp.	100 °C	51.37	74.83	48.26 ^a^	27.60	97.81^a^	101.93 ^a^	103.70 ^a^	100.00 ^a^	88.11
	140 °C	37.50	55.00	42.66 ^b^	20.66	81.52 ^b^	90.46 ^b^	90.17 ^b^	85.90 ^b^	85.29
	180 °C	36.28	50.50	45.79 ^a^	25.10	80.96 ^b^	89.58 ^b^	88.69 ^b^	84.96 ^b^	80.54
*p*-value										
Form		<0.001	0.015	0.154	<0.001	<0.001	0.002	0.038	0.397	<0.001
Temp.		<0.001	<0.001	< 0.001	0.002	<0.001	<0.001	<0.001	<0.001	0.069
Form × temp.		<0.001	0.035	0.905	0.001	0.667	0.09	0.110	0.478	0.110
Significant *p*-values for contrasts										
NM 100 °C vs. M 100 °C		<0.001	-	-	<0.001	-	-	-	-	-
NM 140 °C vs. M 140 °C		<0.001	-	-	<0.001	-	-	-	-	-
NM 180 °C vs. M 180 °C		<0.001	<0.001	-	<0.001	-	-	-	-	-
NM 100 °C vs. NM 140 °C		<0.001	<0.001	-	-	-	-	-	-	-
NM. 100 °C vs. NM 180 °C		<0.001	<0.001	-	-	-	-	-	-	-
NM 140 °C vs. NM 18 0 °C		<0.001	0.013	-	0.039	-	-	-	-	-
M 100 °C vs. M 140 °C		<0.001	0.011	-	<0.001	-	-	-	-	-
M 100 °C vs. M. 180 °C		<0.001	0.037	-	<0.001	-	-	-	-	-
M 140 °C vs. M 180 °C		-	-	-		-	-	-	-	-

^1^ Main effects are shown for responses in which the interaction was not significant, whereas contrasts are shown where a significant interaction was detected. VA; vitamin A, VD_3_; vitamin D_3_, VE; vitamin E, VK_3_; vitamin K_3_, VB_1_; vitamin B_1_, VB_2_; vitamin B_2_, VB_3_; vitamin B_3_, VB_5_; vitamin B_5_, VB_6_; vitamin B_6_. ^x, y^ Means within a column that lack a common superscript differ (*p* < 0.05). ^a, b, c^ Means within a column that lack a common superscript differ (*p* < 0.05). NM, non-microencapsulated; M, microencapsulated. The changes in vitamin concentration during extrusion are presented in Table S1.

**Table 6:** Effects of pelleting parameters and vitamin forms (non-microencapsulated or microencapsulated) on the percentage of vitamins in diets (Experiment 2) ^1^.

Form	Processing	VA	VD_3_	VE	VK_3_	VB_1_	VB_2_	VB_3_	VB_5_	VB_6_
NM	LTLR	94.29	90.03	97.38	31.74	78.58	96.27	94.19	97.77	91.01
	LTHR	93.25	84.42	95.15	27.74	76.05	94.69	92.92	95.54	96.92
	HTLR	92.57	87.81	97.54	29.69	82.21	96.48	96.80	97.22	96.61
	HTHR	88.57	88.71	95.76	23.49	81.52	96.78	96.13	95.30	96.56
M	LTLR	98.11	89.90	97.61	38.43	90.04	96.03	94.54	97.06	90.70
	LTHR	96.12	91.62	97.28	32.91	89.59	96.27	94.13	98.61	89.69
	HTLR	96.48	88.87	96.18	39.44	85.60	98.00	95.75	95.96	94.97
	HTHR	95.42	87.53	95.95	30.95	92.39	96.66	94.65	95.96	95.16
SEM		1.76	4.64	2.43	1.32	1.73	0.73	1.15	2.07	2.69
Main effects										
Form	NM	92.17 ^y^	87.74	96.46	28.16 ^y^	79.59 ^y^	96.05	95.01	96.46	95.27
	M	96.53 ^x^	89.48	96.75	35.43 ^x^	89.41 ^x^	96.74	94.77	96.90	92.63
Processing	LTLR	96.20	89.97	97.50	35.08 ^a^	84.31	96.15	94.37	97.41	90.85
	LTHR	94.69	88.02	96.86	30.33 ^b^	82.82	95.48	93.52	97.07	93.30
	HTLR	94.53	88.34	96.21	34.57 ^a^	83.91	97.24	96.28	96.59	95.79
	HTHR	92.00	88.12	95.85	27.22 ^b^	86.96	96.72	95.39	95.63	95.86
*p*-value										
Form		0.001	0.599	0.864	< 0.001	< 0.001	0.231	0.778	0.766	0.592
Processing		0.135	0.972	0.911	< 0.001	0.164	0.169	0.111	0.839	0.105
Form × processing		0.697	0.806	0.914	0.386	0.135	0.495	0.672	0.729	0.440

^1^ NM, non-microencapsulated; M, microencapsulated; L:D ratio, pellet mill die length-to-diameter ratio; LTLR, low temperature + low L:D ratio; LTHR, low temperature + high L:D ratio; HTLR, high temperature + low L:D ratio; HTHR, high temperature + high L:D ratio. VA; vitamin A, VD_3_; vitamin D_3_, VE; vitamin E, VK_3_; vitamin K_3_, VB_1_; vitamin B_1_, VB_2_; vitamin B_2_, VB_3_; vitamin B_3_, VB_5_; vitamin B_5_, VB_6_; vitamin B_6_. ^x, y^ Means within a column that lack a common superscript differ (*p* < 0.05). ^a, b^ Means within a column that lack a common superscript differ (*p* < 0.05). The changes in vitamin concentration during pelleting are presented in Table S2.

**Table 7:** Comparison and ranking of activity loss of non-microencapsulated vitamins in extrusion and pelleting ^1^.

Item	Extrusion			Overall Rank in Extrusion	Pelleting ^2^				Overall Rank in Pelleting	Overall Rank
	100 °C	140 °C	180 °C		LTLR	LTHR	HTLR	HTHR		
VA	2	2	2	2	6	5	4	3	4	2
VD_3_	4	4	4	4	3	3	3	4	3	3
VE	3	3	3	3	8	7	8	6	7	5
VK_3_	1	1	1	1	1	1	1	1	1	1
VB_1_	6	6	6	6	2	2	2	2	2	4
VB_2_	7	8	8	8	7	6	9	9	8	8
VB_3_	9	9	9	9	5	4	6	7	5	7
VB_5_	8	7	7	7	9	8	7	5	7	7
VB_6_	5	5	5	5	4	9	5	8	6	6

^1^ Based on the percent of commercial vitamin loss and vitamin recovery ranked method reported by Shurson et al. [20]. Vitamins that exhibited the highest % loss in three extrusions or four pelleting were, respectively, ranked 1, whereas vitamins with the lowest activity loss in this processing were separately ranked 9. Overall, the rank in the extrusion is according to mean of the three ranks, and the overall rank in pelleting is according to the mean of the four ranks. Overall, the rank is according to mean of the ranks in the extrusion and pelleting. VA; vitamin A, VD_3_; vitamin D_3_, VE; vitamin E, VK_3_; vitamin K_3_, VB_1_; vitamin B_1_, VB_2_; vitamin B_2_, VB_3_; vitamin B_3_, VB_5_; vitamin B_5_, VB_6_; vitamin B_6_. ^2^ L:D ratio, pellet mill die length-to-diameter ratio; LTLR, low temperature + low L:D ratio; LTHR, low temperature + high L:D ratio; HTLR, high temperature + low L:D ratio; HTHR, high temperature + high L:D ratio.

**Table 8:** Comparison and ranking of activity loss of microencapsulated vitamins in extrusion and pelleting ^1^.

Item	Extrusion			Overall Rank in Extrusion	Pelleting ^2^				Overall Rank in Pelleting	Overall Rank
	100 °C	140 °C	180 °C		LTLR	LTHR	HTLR	HTHR		
VA	3	2	2	2	9	6	8	6	6	3
VD_3_	4	4	4	4	3	4	3	2	3	2
VE	2	3	3	3	8	8	7	7	7	4
VK_3_	1	1	1	1	1	1	1	1	1	1
VB_1_	5	5	5	5	2	2	2	3	2	2
VB_2_	9	8	9	8	6	7	9	9	9	8
VB_3_	8	7	8	7	5	5	5	4	5	6
VB_5_	6	6	6	6	7	9	6	8	8	7
VB_6_	7	9	7	7	4	3	4	5	4	5

^1^ Based on the percent of commercial vitamin loss and vitamin recovery ranked method reported by Shurson et al. [20]. Vitamins that exhibited the highest % loss in three extrusions or four pelleting were, respectively, ranked 1, whereas vitamins with the lowest activity loss in this processing were separately ranked 9. Overall, the rank in the extrusion is according to mean of the three ranks, and the overall rank in pelleting is according to the mean of the four ranks. Overall, the rank is according to mean of the ranks in the extrusion and pelleting. VA; vitamin A, VD_3_; vitamin D_3_, VE; vitamin E, VK_3_; vitamin K_3_, VB_1_; vitamin B_1_, VB_2_; vitamin B_2_, VB_3_; vitamin B_3_, VB_5_; vitamin B_5_, VB_6_; vitamin B_6_. ^2^ L:D ratio, pellet mill die length-to-diameter ratio; LTLR, low temperature + low L:D ratio; LTHR, low temperature + high L:D ratio; HTLR, high temperature + low L:D ratio; HTHR, high temperature + high L:D ratio.

## Supplementary Materials

The following are available online at https://www.mdpi.com/2076-2615/10/5/894/s1, Table S1: Effects of extruded temperature (Temp.) and vitamin forms (non-microencapsulated or microencapsulated) on concentrations of vitamin in diets (Dry matter basis, Experiment 1), Table S2: Effects of pelleting parameters and vitamin forms (non-microencapsulated or microencapsulated) on the concentration of vitamins in diets (Dry matter basis, Experiment 2).
