# Supplementary material for: Evaluation of Extrusion Temperatures, Pelleting Parameters, and Vitamin Forms on Vitamin Stability in Feed
Source: Animals (Basel). 2020 May 20;10(5):894. doi: 10.3390/ani10050894 (PMC7278472; doi:10.3390/ani10050894)
Supplement: Supplementary file 1 [file animals-10-00894-s001.pdf]

Supplementary Material Table S1: Effects of extruded temperature (Temp.) and vitamin forms (non-microencapsulated or microencapsulated) on concentrations of vitamin in diets (Dry matter basis, Experiment 1) [1].

Form	NM			M		
**Temp.**	**100°C**	**140°C**	**180°C**	**100°C**	**140°C**	**180°C**
VA, IU/kg	6330.08	4746.88	4201.84	7775.56	5548.06	5761.72
VD_3, IU/kg	2280.51	1638.98	1241.73	2359.25	1771.39	1892.26
VE, mg/kg	15.21	13.56	14.51	15.29	13.40	14.43
VK_3, mg/kg	0.22	0.19	0.36	1.53	1.12	1.23
VB_1, mg/kg	2.89	2.42	2.39	3.04	2.53	2.52
VB_2, mg/kg	6.04	5.37	5.52	6.43	5.58	5.55
VB_3, mg/kg	31.20	27.29	26.51	32.34	27.96	27.84
VB_5, mg/kg	18.27	15.53	15.75	18.45	16.01	15.45
VB_6, mg/kg	2.30	2.42	2.23	3.21	2.91	2.81

[1] VA; vitamin A, VD_3; vitamin D_3, VE; vitamin E, VK_3; vitamin K_3, VB_1; vitamin B_1, VB_2; vitamin B_2, VB_3; vitamin B_3, VB_5; vitamin B_5, VB_6; vitamin B_6. NM, non-microencapsulated; M, microencapsulated.

Supplementary Material Table S2: Effects of pelleting parameters and vitamin forms (non-microencapsulated or microencapsulated) on concentration of vitamins in diets (Dry matter basis, Experiment 2) [1]

Form	NM				M			
**Processing**	**LTLR**	**LTHR**	**HTLR**	**HTHR**	**LTLR**	**LTHR**	**HTLR**	**HTHR**
VA, IU/kg	12880.09	12738.02	12645.14	12098.73	13523.49	13249.19	13298.81	13152.70
VD_3, IU/kg	2776.77	2603.74	2708.30	2736.06	2800.70	2854.28	2768.61	2726.87
VE, mg/kg	31.28	30.56	31.33	30.76	30.36	30.25	29.91	29.84
VK_3, mg/kg	0.99	0.87	0.93	0.73	1.23	1.05	1.26	0.99
VB_1, mg/kg	2.40	2.32	2.51	2.49	2.72	2.71	2.59	2.79
VB_2, mg/kg	5.89	5.80	5.90	5.92	5.87	5.88	5.99	5.91
VB_3, mg/kg	28.64	28.26	29.44	29.23	29.18	29.05	29.55	29.21
VB_5, mg/kg	18.01	17.60	17.91	17.55	17.76	18.05	17.56	17.56
VB_6, mg/kg	2.82	3.00	2.99	2.99	2.86	2.83	2.99	3.00

[1] NM, non-microencapsulated; M, microencapsulated; L:D ratio, pellet mill die length-to-diameter ratio; LTLR, low temperature + low L:D ratio; LTHR, low temperature + high L:D ratio; HTLR, high temperature + low L:D ratio; HTHR, high temperature + high L:D ratio. VA; vitamin A, VD_3; vitamin D_3, VE; vitamin E, VK_3; vitamin K_3, VB_1; vitamin B_1, VB_2; vitamin B_2, VB_3; vitamin B_3, VB_5; vitamin B_5, VB_6; vitamin B_6.
